# Comparison of the amount of artifacts induced by zirconium and titanium implants in cone-beam computed tomography images

**DOI:** 10.1186/s12880-022-00884-5

**Published:** 2022-09-03

**Authors:** Abbas Shokri, Fariborz Vafaee, Leila Haghighat, Shiva Shahabi, Maryam Farhadian, Mohammad Reza Jamalpour

**Affiliations:** 1 Department of Oral and Maxillofacial Radiology, Dental Implants Research Center, Hamadan University of Medical Sciences, Hamadan, Iran; 2 Department of Prosthodontics, Dental Implants Research Center, Hamadan University of Medical Sciences, Hamadan, Iran; 3 Shiraz, Iran; 4 Dental Implants Research Center, Hamadan University of Medical Sciences, Hamadan, Iran; 5 Department of Biostatistics, School of Public Health and Research Center for Health Sciences, Hamadan University of Medical Sciences, Hamadan, Iran; 6 Department of Oral and Maxillofacial Surgery, Dental Implants Research Center, Hamadan University of Medical Sciences, Shaheed Fahmideh Avenue, Hamadan, 6517838636 Iran

**Keywords:** Artifacts, Cone-beam computed tomography, Titanium, Zirconium

## Abstract

**Background:**

This study aimed to compare the amount of artifacts induced by the titanium and zirconium implants on cone-beam computed tomography (CBCT) and assess the effect of different exposure settings on the image quality for both materials.

**Methods:**

In this experimental study, 30 zirconium and 30 titanium implants were placed in bovine rib bone blocks. CBCT images were taken in two different fields of view (FOV: 4 × 6 cm^2^ and 6 × 8 cm^2^) and at two resolutions (133 µ and 200 µ voxel size). Subsequently, two observers assessed the images and detected the amount of artifacts around the implants through gray values. Data were analyzed by paired *t* test and independent *t* test using SPSS 21 and the 0.05 significance level.

**Results:**

The results showed that titanium implants caused lower amounts of artifacts than zirconium implants, which was statistically significant (*P* < 0.001). The larger FOV (6 × 8 cm^2^) resulted in a lower amount of artifacts in both groups, although the results were only statistically significant in the zirconium group (*P* < 0.001). The amount of artifacts was increased when using the 133 µ voxel size in both groups, which was only significant in the zirconium group (*P* < 0.001).

**Conclusion:**

Our results suggest that zirconium implants induce higher amounts of artifacts than titanium ones. We also concluded that the artifacts could be minimized using the larger FOV and voxel size.

## Introduction

Cone-beam computed tomography (CBCT) has developed as a three-dimensional imaging system used in various specialties of dentistry to provide precise diagnosis and treatment plans [1, 2]. In implantology, preoperative surgery planning and postoperative evaluation are critical steps. CBCT is recommended as a preoperative examination of dental implants [3, 4]. It enables the accurate localization of anatomical structures, quantification of the remaining bone, and exact measurements of both depth and height of the implantation site [5].

Besides the numerous advantages of the CBCT technique, some problems limit its application. The most commonly encountered problem is the formation of image artifacts [6]. Artifacts are visualized structures formed in the image through the data reconstruction process that do not represent the subject being studied [1, 7]. Factors involved in inducing the artifacts can be classified as follows: 1) artifacts caused by the physical structure of the CBCT system, such as the mathematical format used in the 3-dimensional reconstruction, 2) artifacts related to the image acquisition, such as patient movement, exposure settings including milliamperage (mA) and peak kilovoltage (kVp), and presence of high-density elements [5, 8–10].

An X-ray beam is composed of Individual photons with various levels of energy. When the x-ray beam passes through an object, lower-energy photons are absorbed more rapidly than higher-energy ones. Thus, the beam reaching the detector is mainly composed of higher-energy photons. This phenomenon, known as beam hardening, is one of the prominent causes of artifacts, including cupping artifacts and dark bands or streaks [1].

In the process of imaging a cylindrical object, x-rays passing through the middle part of the object are hardened more than those passing through the edges. Since the central part is composed of more material than edges, it can absorb more low-energy photons. The hardened beam that reaches the detectors is less attenuated than expected, and this difference between the attenuation level of the ideal projection and projection with beam hardening results in the cupped shape artifact formation surrounding the cylindrical object [6, 11].

Dark bands or streaks are artifacts that appear in an image between two high-density objects. Due to the rotational position of the tube, beam hardening occurs at different rates leading to the formation of these artifacts. When the beam passes through both objects at a particular tube position, it is more hardened than when it passes through one of the objects at another tube position. This difference in the attenuation level causes streaking artifacts to happen [11, 12].

As one of the high-density materials used in dentistry, traditional titanium implants are accounted for artifact formation in CBCT images, which may prevent proper analysis of the peri-implant area. Therefore, CBCT is not an inerrant protocol for post-implant evaluations due to the inherent limitations of the imaging process [7, 13, 14].

Metal-free implants such as zirconium have recently become a feasible alternative to titanium implants. Zirconium implants are preferable in the esthetic area due to their tooth-like color [15]. Reduced plaque accumulation and accelerated biocompatibility around zirconium implants have diminished the inflammatory and allergic reactions compared to titanium implants [16, 17]. In addition, it has been reported that the mechanical stability and success rate of zirconium and titanium implants are comparable [18–20]. Despite the mentioned advantages, zirconium is also a high-density element causing image artifacts.

Each type of dental implant material impacts the CBCT image quality differently, and exposure settings' alterations affect the image quality depending on the type of implant's material. Therefore, we aimed to compare the amounts of artifacts produced by titanium and zirconium implants on CBCT images. Additionally, we evaluated how different exposure parameters of a CBCT system, such as field of view (FOV) and resolution, impact the image quality for different materials (titanium and zirconium).

## Materials and methods

**Fig. 1:**
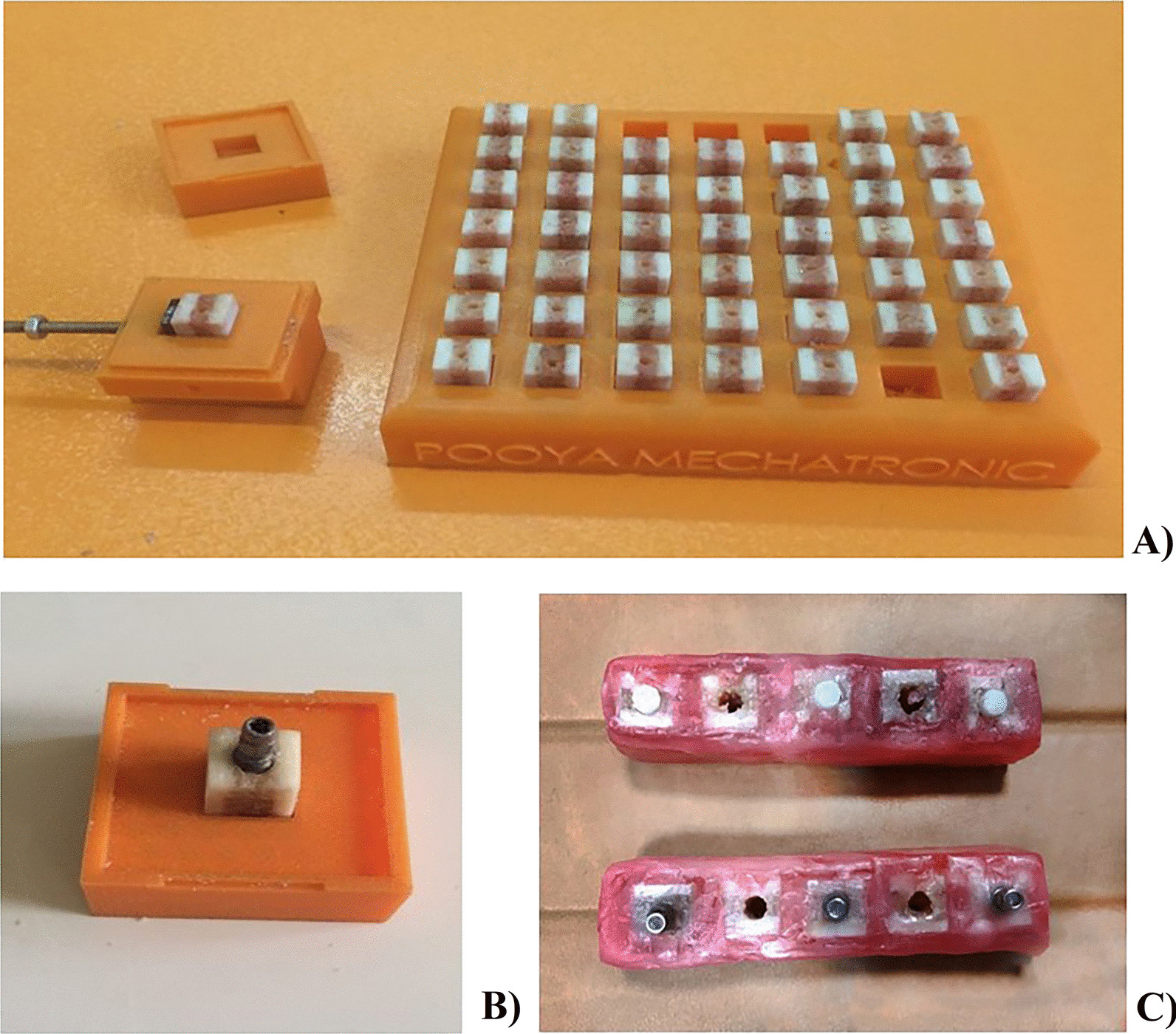
**A** Prepared bone blocks. **B** Bone block with an inserted implant. **C** Implant containing rims prepared for imaging

This experimental study was approved by the Ethics Committee of Hamadan University of Medical Science (IR.UMSHA.REC.1397.544). Bone blocks were prepared using freshly slaughtered cows' rib bones that appropriately resemble the human alveolar bone. The ribs were kept refrigerated to avoid dehydration. After removing the soft tissue around the ribs, we used a computer numerical controlled machine (Pooya Mechatronic, Hamadan, Iran) to cut the bones into 60 bone blocks of exactly equal dimensions, measuring 8 × 8 × 11 mm [21, 22]. A plastic box with 8 × 8 × 11 mm holes was designed (Pooya Mechatronic, Hamadan, Iran). This box helped hold the blocks tightly during implant insertion (Fig. 1A). An experienced oral and maxillofacial surgeon inserted 30 titanium implants (SIC invent AG, Basel, Switzerland) and 30 zirconium implants (Pouyan Teb Hegmataneh Industries Company, Hamadan, Iran) in the bone blocks (Fig. 1B). The zirconium implants were manufactured especially for this study. Titanium and zirconium implants were identical in shape and size (4 × 11 mm). The custom-made zirconium implants were used to eliminate confounding variables, such as length, diameter, and surface properties of the implants, and the type of material was the only variable between the two groups.

Rectangular boxes were fabricated using two layers of 2 mm red dental wax. Bone blocks containing implants and those without implants were placed in these boxes alternatively [23] (Fig. 1C). Each box contained five bone blocks and was subjected to CBCT imaging. The selected FOVs could cover the five blocks of the arranged rims in a single CBCT take.

The CBCT images were obtained using CBCT Cranex 3D (Soredex, Tuusula, Finland) with exposure settings of 90 kVp, 10 mA, 6.1 s, two different sizes of FOVs (4 × 6 cm^2^ and 6 × 8 cm^2^), and two different resolution modes (high: 133 µ voxel size and low: 200 µ voxel size).

All CBCT images were imported as DICOM files into On Demand 3D dental software (Cybermed, Seoul, Korea) to be reconstructed and analyzed. For the assessment of images, cross-sections were reconstructed so that the sections passed through the implant's center.

**Fig. 2:**
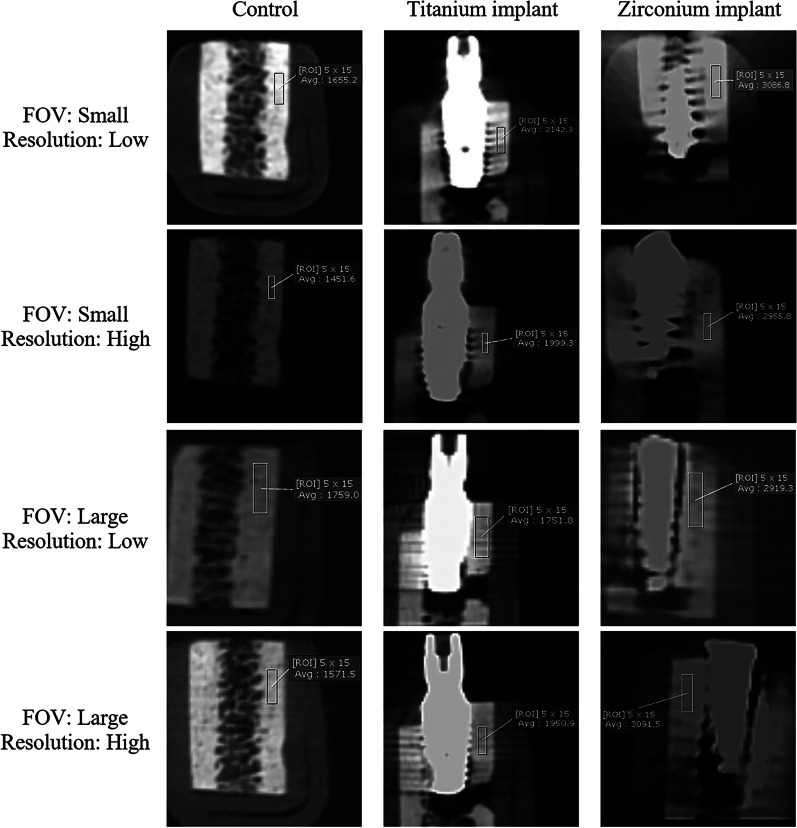
The evaluation of the amount of artifacts in different study groups

The amount of artifacts was evaluated by measuring the difference between the mean gray value of the bone blocks with inserted implants and those without implants. A perpendicular axis to the implant's longitudinal axis was reconstructed for data evaluation like studies of Benic et al. [24] and Sancho-Puchades et al. [25]. The region of interest (ROI) with 5 × 15 pixels dimensions was set for each section. The ROI was placed at the middle of the block buccolingually, covering the highest amount of artifacts from the corono-apical point of view. The ROI box was moved from coronal to apical pixel by pixel, and each box's gray value was measured. Finally, the ROI with the highest recorded gray value was selected, presenting the amount of artifacts (Fig. 2).

Two observers (one maxillofacial radiologist and one dentist) examined all samples' scans twice at two-week intervals. Agreement between the two observers was assessed.

Data were analyzed using SPSS software version 21. Data were reported as mean ± standard deviation and were analyzed using paired t-test and independent t-test. The intra-class correlation coefficient (ICC) was used to control inter-observer and intra-observer agreements. The significance level was set at 0.05.

## Results

The calculated ICCs were 0.961 (intra-observer) and 0.945 (inter-observer), showing excellent agreement between the two observers. Therefore, we reported study results according to the measurements obtained from the first iteration of one of the observer's reports.

**Fig. 3:**
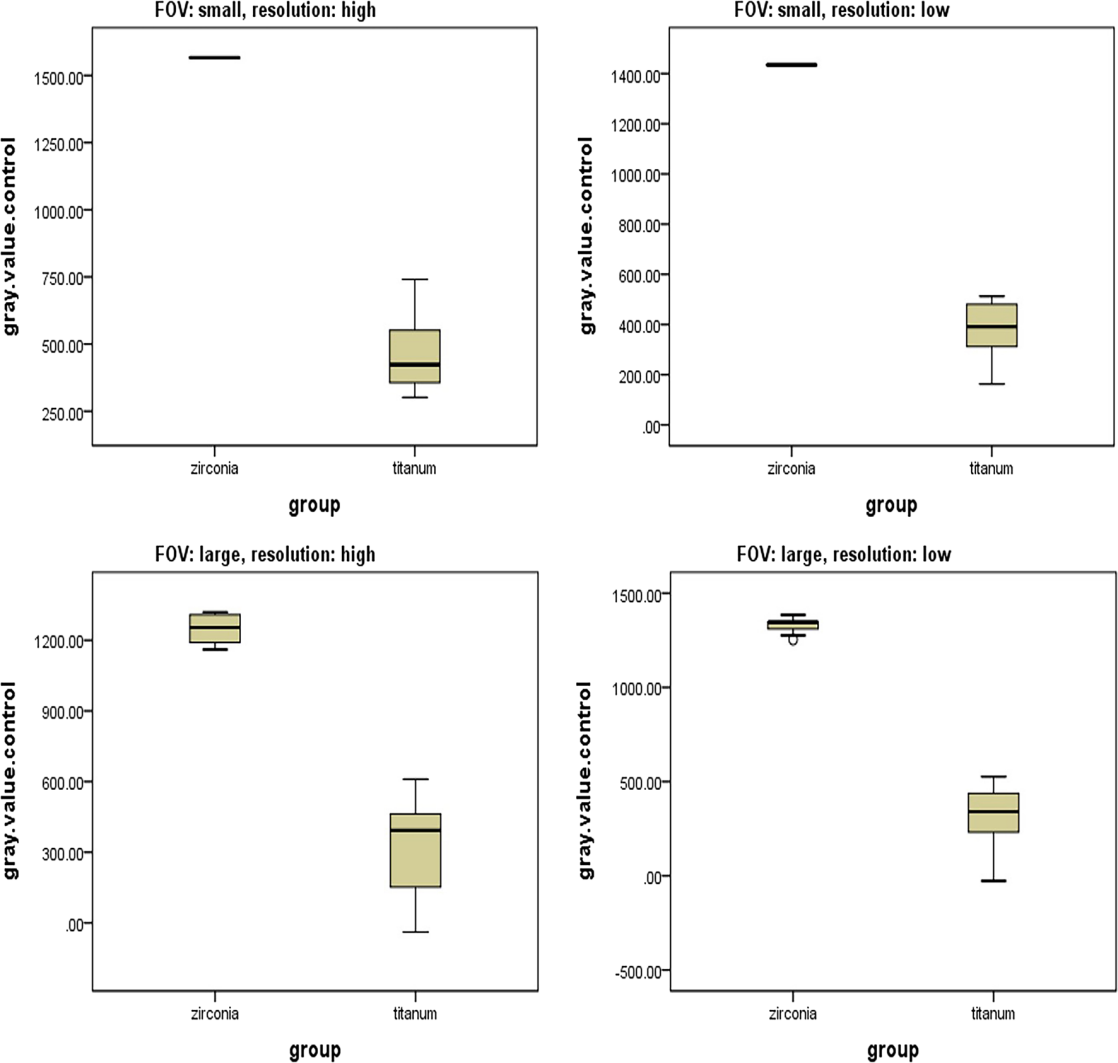
Box diagrams comparing the amount of artifacts induced by zirconium and titanium implants in different fields of view and resolutions

The results showed that the amount of artifacts induced by the zirconium implant group was significantly higher than the titanium implant group (*P* < 0.001). The difference between the two groups of implants was significant in both FOVs (small and large) and both resolutions (high: 133 µ voxel size and low: 200 µ voxel size) (*P* < 0.001, Table 1, Fig. 3).

**Table 1:** Comparison of the amount of artifacts induced by zirconium and titanium implants

FOV	Resolution	Implant’s type	Gray value (mean ± SD)	Mean difference ± SE	*P*-value*
Small	High (133 µ voxel size)	Zirconium	1566.10 ± 0.77	1098.65 ± 41.50	< 0.001
		Titanium	167.44 ± 143.76		
	Low (200 µ voxel size)	Zirconium	1434.46 ± 0.9	1061.18 ± 33.96	< 0.001
		Titanium	373.28 ± 117.65		
Large	High (133 µ voxel size)	Zirconium	1251.02 ± 62.74	928.86 ± 60.71	< 0.001
		Titanium	322.16 ± 200.75		
	Low (200 µ voxel size)	Zirconium	1329.05 ± 36.43	1016.52 ± 49.40	< 0.001
		Titanium	312.53 ± 167.23		

*Independent *t* test, *FOV* field of view, *SD* standard deviation, *SE* standard error

The amount of artifacts induced by zirconium implants was significantly decreased by the increment in the size of the FOV in both high and low resolutions (*P* < 0.001). In the titanium implants group, the amount of artifacts was also decreased by the increment in the size of the FOV in both resolutions, but the results were not statistically significant (high resolution: *P* = 0.212 and low resolution: *P* = 0.281) (Table 2).

**Table 2:** Comparison of the amount of artifacts induced by study groups in large and small FOVs

Implant’s type	Resolution	FOV	Gray value (mean ± SD)	Mean difference ± SE	*P*-value*
Zirconium	High (133 µ voxel size)	Small	1566.10 ± 0.77	315.07 ± 18.11	< 0.001
		Large	1251.02 ± 62.74		
	Low (200 µ voxel size)	Small	1434.46 ± 0.9	105.40 ± 10.52	< 0.001
		Large	1329.05 ± 36.43		
Titanium	High (133 µ voxel size)	Small	467.44 ± 143.7	145.28 ± 71.28	0.212
		Large	322.16 ± 200.75		
	Low (200 µ voxel size)	Small	373.28 ± 117.65	60.75 ± 59.02	0.281
		Large	312.53 ± 167.23		

*Paired *t* test, *FOV* field of view, *SD* standard deviation, *SE* standard error

The amount of artifacts increased with resolution enhancement in both study groups, which was statistically significant only in the zirconium group (*P* < 0.001, Table 3).

**Table 3:** Comparison of the amount of artifacts induced by study groups in high and low resolutions

Implant’s type	Resolution	Gray value (mean ± SD)	Mean difference ± SE	*P*-value*
Zirconium	High (133 µ voxel size)	1408.56 ± 166.67	26.80 ± 36.12	< 0.001
	Low (200 µ voxel size)	1381.76 ± 59.44		
Titanium	High (133 µ voxel size)	394.80 ± 186.18	51.89 ± 48.14	0.573
	Low (200 µ voxel size)	342.90 ± 144.77		

*Paired *t* test, *SD* standard deviation, *SE* standard error

## Discussion

Implant placement aims to restore acceptable esthetic and function without affecting the surrounding soft and hard tissues. Following the implant insertion, patient follow-up is required. In some cases, the CBCT image of the implant is beneficial to complication diagnosis and patient follow-up [26]. Thus, the limitations of the CBCT systems and the amount of artifacts in these images should be considered. However, Chagas et al. [27] found no significant difference regarding diagnostic accuracy between CBCT images of peri-implant bone defects around titanium implants and zirconium dioxide implants.

Numerous studies have evaluated the effect of various parameters, such as FOV, kVp, mA, system type, exposure time, type of material, and surrounding bone, on artifacts by the dental implant in the CBCT images [3, 28, 29]. Though, how we should change the exposure parameters depending on the specific CBCT system or type of implant being used is still a controversial matter. Hence, this study investigated the effect of different exposure parameters and implant materials on the amount of artifacts based on the CBCT system and exposure settings used in our office. Exposure parameters, including FOV and resolution, were studied in the Cranex 3D CBCT system.

Our results showed that the amount of artifacts around zirconium implants was higher than in titanium implants. This outcome confirmed the effect of implant material on the image quality. According to the Mendeleev table, titanium atoms have an atomic number of 22 and a density of 4.506 g.cm^−3^, and zirconium atoms have an atomic number of 40 and a density of 6.511 g.cm^−3^. This difference in atomic numbers and densities of the two implant materials may justify the higher amount of artifacts in zirconium implants.

Shokri et al. [30] examined the effect of different exposure settings in a CBCT system on reducing the metal artifact around dental implants at different bone densities. The results showed that implants induce different amounts of artifacts in CBCT images by altering conditions such as FOV, bone density, time, amperage, and voltage. Notably, the effect of voltage on the amount of artifacts was more than other factors. Therefore, to equalize the conditions and eliminate the mediating factors, we set the same setting (90 kVp and 10 mA) and bone density in all images and conducted the study in two FOVs (4 × 6 cm^2^ and 6 × 8 cm^2^) as well as two resolutions (high: 133 µ voxel size and low: 200 µ voxel size) [31].

Sancho-Puchades et al. [25] compared the artifacts generated by titanium, titanium-zirconium, and zirconium implants in vitro. They inserted implants in 20 bone models of human mandibles and investigated the amount of artifacts in CBCT images (KaVo Dental GmbH, Biberach, Germany). Similar to our observations, they concluded that the amount of artifacts produced by zirconium implants was more considerable than others. It should be mentioned that they used different exposure settings of 120 kVp, 5 mA, and 26 s radiation time.

Fontenele et al. [3] also investigated the amount of artifacts induced by implants inserted in the human mandible at distances of 1.5 cm, 2.5 cm, and 3.5 cm with angles of 65°, 90°, 115°, and 140° by three various CBCT systems (Picasso Trio, ProMax 3D, and 3D Accuitomo 80). Using 80 kVp and 5 mA exposure settings, they concluded that zirconium implants produce the most significant artifacts. This outcome was in line with our study. Observations showed that the difference between the amount of artifacts induced by three different CBCT systems was not statistically significant for titanium implants, while zirconium implants showed significantly different amounts of artifacts depending on the CBCT system.

In 2017, Smeets et al. [26] examined artifacts caused by zirconium, titanium, and titanium-zirconium implants in magnetic resonance imaging (MRI), conventional tomography (CT), and CBCT images using almost identical exposure conditions as ours (90 kVp, 8 mA, 13.6 s). In MRI images, the amount of artifacts from the zirconium implants was the least, while in the CT and CBCT images, the titanium implants produced the minimum amount of artifacts. As a result, they suggested MRI as the excellent choice of imaging for patients with zirconium implants and CT or CBCT for patients with titanium and titanium-zirconium implants. It is mentionable that the advantage of this study was to explore the amount of artifacts in different imaging modalities; nevertheless, it confirmed the results of the present study.

In addition to the implant's material, the present study showed that FOV is one of the factors influencing image quality. FOV size varies based on the system used for imaging, and it should be carefully set so that the resulting image can provide valuable information for the patient's diagnosis and treatment plan. Selecting the appropriate FOV size reduces the patient radiation exposure and enhances the image quality [32].

FOV is defined by the area of interest to be covered by the beam. A pixel is the smallest distinguishable part of an image. In a fixed matrix size, increasing the FOV leads to larger pixel sizes [33]. The pixel size can affect the diagnostic value of an image. Images acquired in smaller pixel sizes may appear sharper due to the higher resolution. However, by using smaller pixel sizes, x-ray photons are less likely to reach the detector, creating more noise [34]. Moreover, reducing the pixel size is not applicable since it prolongs the scanning time resulting in higher radiation exposure and risk of patient movement [35]. So, it is reasonable that larger FOVs present lower amounts of artifacts as a consequence of providing greater pixels.

In the present study, the amount of artifacts around the implants decreased by the FOV expansion. Previous studies have also found FOV as one of the main factors affecting image quality. In agreement with our results, Pauwels et al. [36] suggested that the large FOV performed better than the small one using the 3D Accuitomo 170 system (90 kVp, 5 mA) for imaging.

Shokri et al. [37] studied the effect of FOV size on gray values in CBCT images. They implanted 4 acrylic cylinders in acrylic phantoms, each containing various materials used in maxillary grafts, including Nanobone, Cenobone, Cerabone, and water (as a control group). CBCT images were taken using 90 kVp, 5 mA, and two different FOVs (4 × 6 cm^2^ and 6 × 8 cm^2^). The results showed that FOV significantly affects the quality of images. The smaller FOV resulted in more variability in gray values and thus increased amounts of artifacts, which was consistent with the current study.

Several factors contribute to the quality of a CBCT image, such as kVp, mA, and the type of the CBCT device [38–40]. These factors widely varied among studies that have evaluated the effect of different FOVs on the amount of artifacts. Thus, comparing the results of these studies could be hindered by the differences in the mentioned contributing factors among studies.

In 2013, Parsa et al. [32] examined the CBCT parameters (FOV, spatial resolution, number of projections, exposure time, and dose selection) on the gray value measurement in the implant area. This study included two CBCT systems (Accuitomo 170 and NewTom 5G) and Multislice CT (MSCT) for imaging. In both CBCT systems, selective spatial resolution and FOV significantly affected gray value measurements. The results presented more significant artifacts with FOV increment by Accuitomo 170 (90 kVp and 5 mA), which was not in line with the present study. This conflict might have resulted from image reconstruction and post-processing differences between the two studies. However, the results obtained from the NewTom 5G (110 kVp and 0.57 mA) showed that the amount of artifacts decreased with FOV increment, which is consistent with our study.

Regarding the effects of resolution alterations (high or low voxel size) on imaging quality, we observed more artifacts with the smaller voxel size in both study groups, but the results were only statistically significant for the zirconium implants group. Therefore, using the larger voxel size seems to benefit the quality improvement of CBCT images from zirconium implants.

Resolution is the ability to detect small details on images depending on the digital systems' voxel size. According to Shokri et al. [41], smaller voxel sizes can increase the resolution. Voxel size is a critical factor that affects the quality and duration of CBCT image reconstruction [42]. Theoretically, as the voxel size decreases, the detector's radiation-sensitive surface decreases, resulting in higher image noise levels. Consequently, there is a need to increase voltage, amperage, or radiation time to improve image accuracy. Thus, reducing the voxel size increases the image's resolution at the cost of additional image noise and patient exposure [43].

Parsa et al. [32] showed that higher resolutions lead to fewer artifacts using the NewTom 5G CBCT system. This inconsistent result with ours might be due to the differences in the systems' spatial resolution used for imaging.

In conclusion, the amount of artifacts induced by dental implants is an inevitable factor affecting the quality of CBCT images. Although, it could be diminished by enhanced precision in interpreting images, improved accuracy in choosing the type of implant, and more attention to imaging settings such as FOV and resolution. Within the limitations of this study, when using CBCT Cranex 3D with exposure settings of 90 kVp, 10 mA, and 6.1 s for evaluating complications after implant insertions, increasing the voxel size would help minimize the artifacts and reach a better diagnosis. Furthermore, zirconium implants induce a higher amount of artifacts than titanium ones.

## Data Availability

The datasets used and/or analyzed during the current study are available from the corresponding author on reasonable request.
